# Supplementary material for: A high-current hydrogel generator with engineered mechanoionic asymmetry
Source: Nat Commun. 2024 Feb 19;15:1494. doi: 10.1038/s41467-024-45931-7 (PMC10876576; doi:10.1038/s41467-024-45931-7)
Supplement: Supplementary file 1 — Supplementary information [file 41467_2024_45931_MOESM1_ESM.pdf]

Supplementary Information

A high-current hydrogel generator with engineered mechanoionic asymmetry

Hongzhen Liu[1], Xianglin Ji[2,3], Zihao Guo[4], Xi Wei[1], Jinchen Fan[5], Peng Shi[2,3], Xiong Pu[4]✉, Feng Gong[6]✉, and Lizhi Xu[1,7]✉

[1]Department of Mechanical Engineering, The University of Hong Kong, Hong Kong SAR, China.

[2]Department of Biomedical Engineering, City University of Hong Kong, Hong Kong SAR, China.

[3]Hong Kong Centre for Cerebro-Cardiovascular Health Engineering, Hong Kong Science Park, Hong Kong SAR, China.

[4]Beijing Institute of Nanoenergy and Nanosystems, Chinese Academy of Sciences, Beijing, China.

[5]School of Materials and Chemistry, University of Shanghai for Science and Technology, Shanghai, PR China

[6]Key Laboratory of Energy Thermal Conversion and Control of Ministry of Education, School of Energy and Environment, Southeast University, Nanjing, China

[7]Advanced Biomedical Instrumentation Centre, Hong Kong Science Park, Shatin, New Territories, Hong Kong SAR, China.

✉e-mail: xulizhi@hku.hk (L.X) puxiong@binn.cas.cn (X.P) gongfeng@seu.edu.cn (F.G)

Content List:

1, Supplementary Note

2, Supplementary Figures

3, Supplementary Tables

4, References

Supplementary Note 1

Theoretical analysis of the mechanoionic hydrogel generator

With compressive strain applied to the hydrogel, the concentration of both anions and cations will increase. Fick's law defines the diffusion flux of ions due to concentration gradient:

$$J_s = -D_s \nabla c_s \quad \text{S1}$$

where J_s (mol m^{-2} s^{-1}) is the diffusion flux of ion s, D_s (m^2 s^{-1}) is the diffusion coefficient of s, and c_s (mol m^{-3}) is the concentration of s. Given that those rich cations are adsorbed by the ACC, aggregating at the interface of the working electrode and electrolyte, the concentration of diffusible cations decreases near the interface in the hydrogel, reducing ∇c_s. For anions, negative adsorption energy leads to the opposite result, increasing ∇c_s. Thus, the net diffusion flux of anions is achieved, which can be regarded as a net ion current:

$$I_{net} = J_{net} z_{Cl^-} F \quad \text{S2}$$

I_{net} (A m^{-2}) is the current density. J_{net} (mol m^{-2} s^{-1}) is the net diffusion flux. z_{Cl^-} is the charge of Cl^-. F (C mol^{-1}) is Faraday's constant.

The thermodynamic diffusion theory was employed to analyze the energy conversion from chemical gradient to electrical energy[1]. The loss in Gibbs free energy (dG) and the converted electric energy (dW) in an infinitesimal time unit (dt) caused by the diffusion of ions are described as follows.

The change in the Gibbs free energy dG caused by the diffusion of ions from the high-strain area (H) to the low-strain area (L) can be written as:

$$\begin{aligned} dG &= dG_H + dG_L \\ &= (\mu_{+H} - \mu_{+L}) dn_{+H} + (\mu_{-H} - \mu_{-L}) dn_{-H} \end{aligned}, += Li^+;\ -= Cl^- \quad \text{S3}$$

$\mu_{+/-}$ is the chemical potential of cations and anions. $dn_{+/-}$ is the change in ion number during the diffusion process. For Li^+ and Cl^-, $dn_{+/-}$ can be calculated as:

$$dn_{+H} = -\frac{|I_+|}{F} dt;\ dn_{-H} = -\frac{|I_-|}{F} dt \quad \text{S4}$$

Where $I_{+/-}$ is the ion current of cations and anions, and F is Faraday's constant.

The chemical potential can be expressed as:

$$\mu = \mu_0 + RT \ln \alpha \quad \text{S5}$$

α is the chemical activity of ions. Therefore,

$$\mu_{+H} - \mu_{+L} = RT \ln\frac{\alpha_{+H}}{\alpha_{+L}},\ \mu_{-H} - \mu_{-L} = RT \ln\frac{\alpha_{-H}}{\alpha_{-L}} \qquad \text{S6}$$

H and L mean the high-strain areas and the low-strain areas. For this device, the concentration of ions in the high-strain area changes with applied pressure, not a stable state. So α_{+H} and α_{-H} are functions of time. S3 could be written as:

$$dG = -\frac{RF}{T}\left(|I_+| \ln\frac{\alpha_{+H}}{\alpha_{+L}} + |I_-| \ln\frac{\alpha_{-H}}{\alpha_{-L}}\right) dt \qquad \text{S7}$$

The converted electrical energy dW can be defined as:

$$dW = I_{net}\left|\varepsilon_j\right| dt \qquad \text{S8}$$

Here I_{net} is the net current as mentioned in S2, and ε_j is the liquid junction potential. This value can be calculated by:

$$\varepsilon_j = \frac{RT}{F}(t_+ \ln\frac{\alpha_{+H}}{\alpha_{+L}} - t_- \ln\frac{\alpha_{-H}}{\alpha_{-L}}) dt \qquad \text{S9}$$

Here, the transference number $t_+ = \frac{|I_+|}{|I_+|+|I_-|};\ t_- = \frac{|I_-|}{|I_+|+|I_-|}$.

Thus,

$$dW = \frac{RT}{F}\left|\frac{|I_-|-|I_+|}{|I_+|+|I_-|}\right| \left|\left(|I_+| \ln\frac{\alpha_{+H}}{\alpha_{+L}} - |I_-| \ln\frac{\alpha_{-H}}{\alpha_{-L}}\right)\right| dt \qquad \text{S10}$$

The energy conversion efficiency from chemical gradient to electrical energy can be calculated by:

$$\eta = \frac{dW}{dG} = \left|\frac{|I_-|-|I_+|}{|I_+|+|I_-|}\right| \frac{\left||I_+| \ln\frac{\alpha_{+H}}{\alpha_{+L}} - |I_-| \ln\frac{\alpha_{-H}}{\alpha_{-L}}\right|}{|I_+| \ln\frac{\alpha_{+H}}{\alpha_{+L}} + |I_-| \ln\frac{\alpha_{-H}}{\alpha_{-L}}} \qquad \text{S11}$$

The selective adsorption induces the separation of anions and cations at the surface between the activated carbon cloth and the hydrogel. This equation indicates that increasing the asymmetry of the diffusion flux is an effective method to increase efficiency, which was verified by Fig. 2b and Fig. 2f .

When the transferred charge is 500 μC, the transferred electrons are:

$$\frac{500\times10^{-6}\times6.24\times10^{18}}{6.02\times10^{23}}\ mol = 5.2 \times 10^{-9}\ mol \qquad \text{S12}$$

Given that $z_{Cl^-} = z_{Li^+} = 1$, to achieve this value, the net diffusion of chloride ions is

5.2×10^{-9} mole.

Assuming that a single quadrangular pyramid unit (4×4×3 mm) is compressed to a truncated square pyramid (4×4×1.5 mm), the changed volume is a small quadrangular pyramid (2×2×1.5 mm). It is known that the device contains 25 pyramid units, and the concentration of the electrolyte is 1 M LiCl. Assuming that only ions in this completely compressed area are affected and ignoring the deformation of the other area of the hydrogel, which is not entirely compressed, the influenced chloride ions are calculated to be:

$$\frac{2\times2\times1.5}{3} \times 25\ mm^3 \times 1\ \frac{mol}{L} = 5 \times 10^{-5}\ mol \quad \text{S13}$$

The ratio between the asymmetric diffusion part to the total influenced ions is:

$$\frac{5.2\times10^{-9}\ mol}{5\times10^{-5}\ mol} = 0.01\% \quad \text{S14}$$

A slight deformation provides considerable ion transfer in the hydrogel. Thus, improving the selective ion adsorption by matching the electrolyte and electrodes may be a more effective method to improve the response currents further.

Supplementary Note 2

Estimated mechanical-electricity conversion efficiency: We calculated the mechanical-electricity conversion efficiency according to the following equations.

$$E_M = \int F dx \qquad \text{S15}$$

Here E_M means the input mechanical energy; F means the force during the compression process; x means the displacement of the ACC electrode.

$$E_E = R \int I^2 dt \qquad \text{S16}$$

Here E_E means the output electrical energy; R is the internal resistance (122 Ω); I is the current and t is the time.

$$\eta = \frac{E_M}{E_E} \times 100\% \qquad \text{S17}$$

Based on the force-displacement curve and current-time curve of the CC-PVA 4M LiCl-ACC device under an applied pressure of 85 kPa, we have calculated that the energy conversion efficiency of the device is approximately 5%.

Supplementary Figures

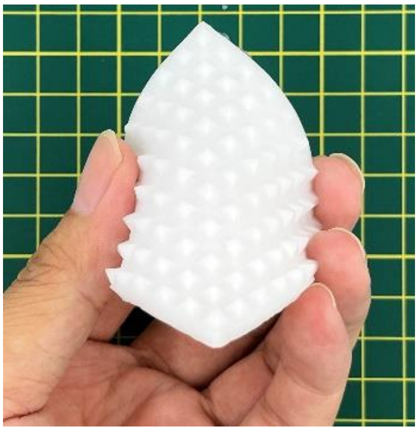

Supplementary Fig. 1 Digital photo of a large area PVA hydrogel with pyramid structure.

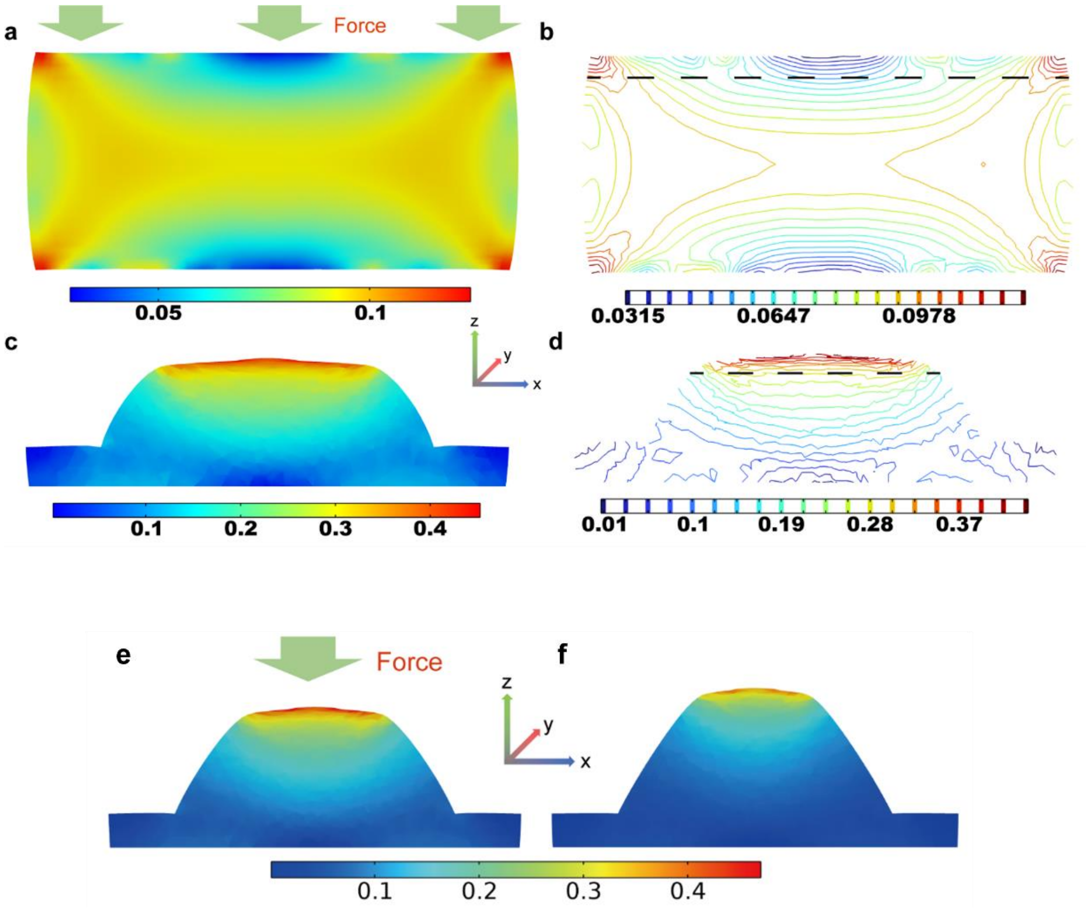

Supplementary Fig. 2 FEA results of compressive strain in center sections of cubic PVA hydrogel (**a** for heatmap and **b** for contour map) and patterned PVA hydrogel (**c** for heatmap and **d** for contour map). The direction of the force is from top to bottom. For the pyramid structure, we used the area of the top of the model after compression deformation, because the geometry parameters of this model changed significantly under compression. Under similar pressure (32 kPa for **a** and 30 kPa for **c**), the strain contours clearly show that the hydrogel with pyramidal structure has significantly enhanced strain gradient. In addition, it can be seen from the cross-sectional view that in the square structure, the strain is symmetrically distributed, with high strain around the edges and low strain in the middle. Since ions tend to diffuse from the high-strain area to the low-strain area, the symmetrical structure may cause the ion flows to cancel each other, reducing the output current. The strain gradient generated by the pyramid structure is from top to bottom, which is more conducive to generating directional ion flow. **e-f**), FEA results of compressive strain in hydrogels with different compressive moduli (60 kPa for **e** and 100 kPa for **f**) under the same external force (~28 mN). A lower compression modulus

may lead to a higher strain gradient, which would generally result in a higher output current. However, other chemical and physical factors may couple with the mechanical properties and contribute the complex electrical responses.

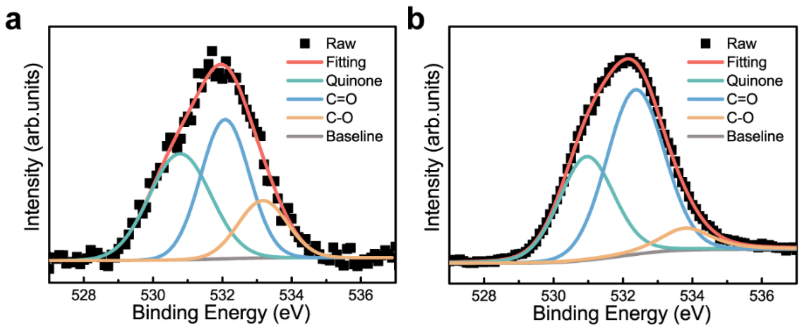

Supplementary Fig. 3 O*1s* XPS spectrums of **a,** CC and **b,** ACC. Both of them show three types of peaks[2]: quinone peak (530.8 eV), C=O (532.2 eV) peak, and C-O peak (533.4 eV).

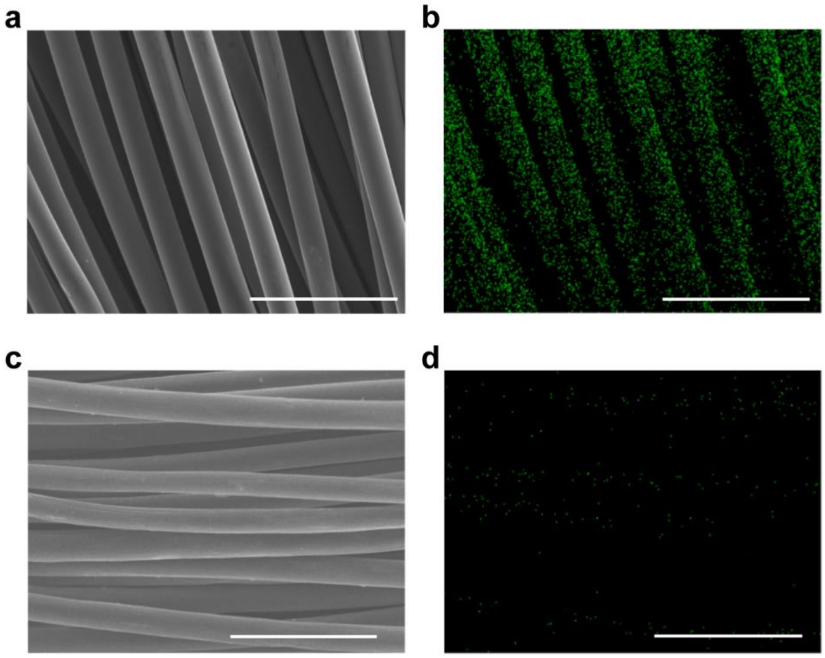

Supplementary Fig. 4 SEM (left) and EDS mapping (right) for oxygen of ACC (**a, b**) and CC (**c, d**). Scale bar: 50 μm. Before and after activation, the morphology of carbon fibers remained almost unchanged, but the oxygen content increased significantly.

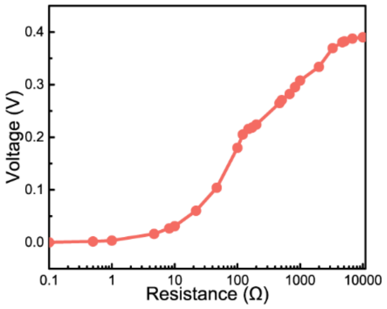

Supplementary Fig. 5 Voltages of the hydrogel generator under various external resistance.

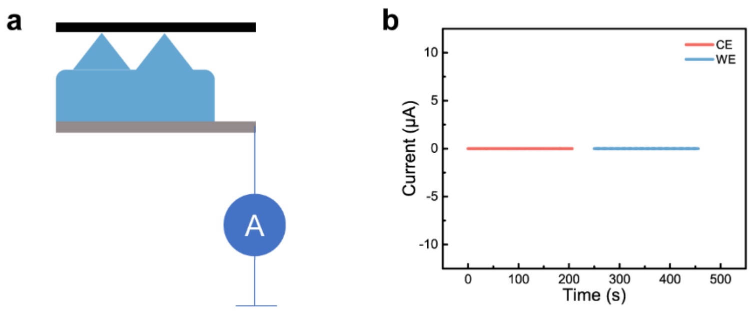

Supplementary Fig. 6 Triboelectric performance of the device. **a**, A schematic of the device. **b**, Output characteristics. The top electrode was subject to pressure-separation cycles. The CE line refers to the output currents between the counter electrode and the ground, and the WE line refers to the output currents between the working electrode and the ground. The extremely low current indicates that the high current does not originate from the triboelectric effect.

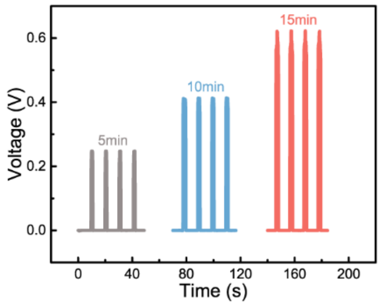

Supplementary Fig. 7 Open-circuit voltages when using ACC with different activation times as the working electrode. Longer activation time brings higher V_{OC}.

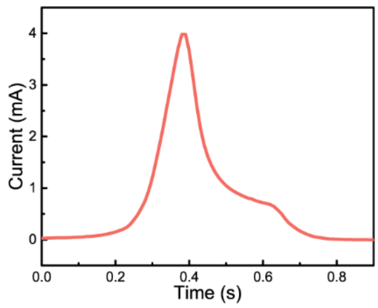

Supplementary Fig. 8 Generated current as a function of time during a standard compression process, which also represents the characteristics of instantaneous ion flux.

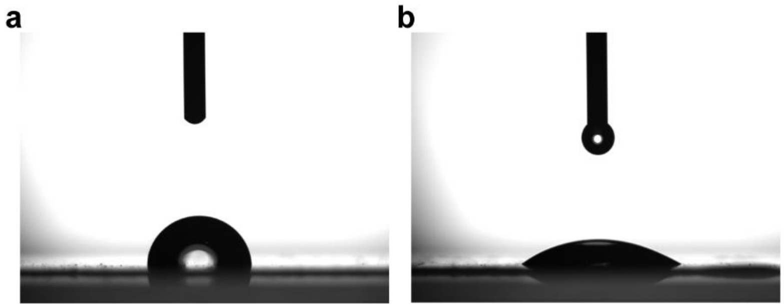

Supplementary Fig. 9 Contact angles of the steel foil before (**a,** 91°) and after (**b,** 32°) plasma treatment. The lower contact angle after plasma treatment indicates the introduction of oxygen-containing functional groups.

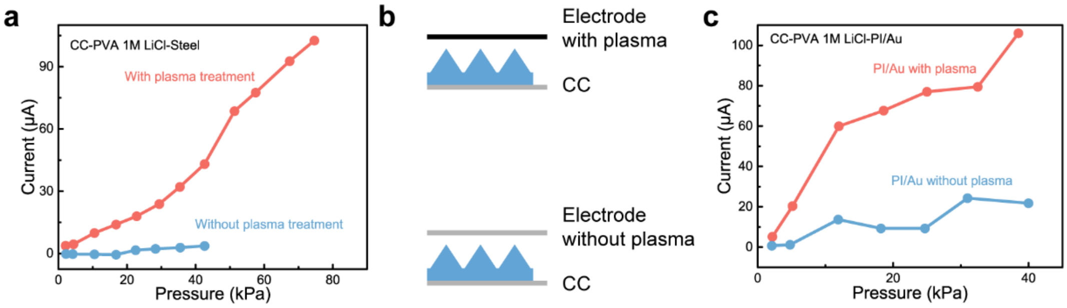

Supplementary Fig. 10 **a,** Current output of devices, in which steel foil with (red line) or without (blue line) plasma treatment serves as the working electrode. **b,** Schematics of the device configuration. **c,** Current output of devices, in which PI/Au film with (red line) or without (blue line) plasma treatment serves as the working electrode. After plasma treatment, the output current increased significantly in both cases. This result is in good agreement with the results mentioned in Fig. 2a when ACC and CC were used as the working electrode, showing the importance of oxygen-containing functional groups.

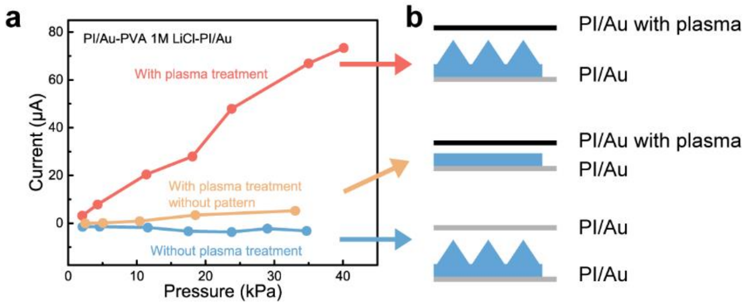

Supplementary Fig. 11 **a,** Response current as a function of applied pressure for various devices involving PI/Au electrodes. The yellow line refers to the pressure-response characteristics using the cubic PVA hydrogel. **b,** Schematic images of corresponding device configurations. The tremendous difference in response currents before and after plasma treatment also shows that oxygen-containing functional groups are vitally important. Meanwhile, the difference between the yellow and red lines proves that the engineered pyramid structure also plays a critical role in improving the current. We chose the PI/Au film because it is very flat and clean, which can significantly reduce the influence of undesired surface deformation of the hydrogel. The value of the blue line is below zero. A possible reason is that when using carbon electrodes, cations may be preferentially adsorbed over anions due to the cation-π interactions, which is in contrast to the metal-electrolyte interface, where the cation-π interactions are absent and anion adsorption may dominate[3]. Thus, the direction of the current is reversed.

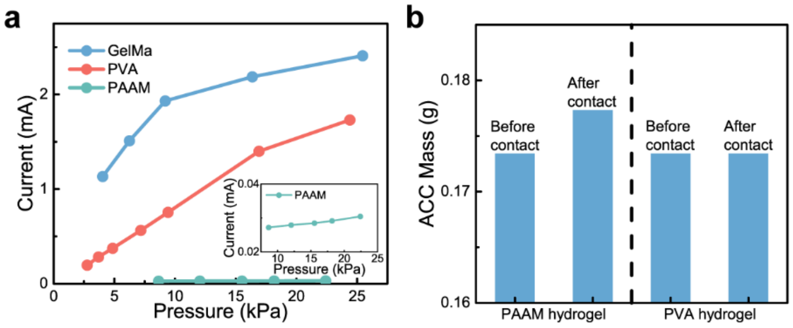

Supplementary Fig. 12 Current outputs of different hydrogels. **a**, Current output for devices based on GelMa, PVA, and PAAM. **b**, Change in mass before and after the contact. Three different hydrogels were tested, i.e. PVA, PAAM, and GelMa hydrogels. All the hydrogels were soaked in 1M LiCl solution and wiped before testing. The PAAM hydrogel was prepared with 33 wt% AAM solution mixed with 0.1 wt% MBAA and 0.5 wt% photo-initiator 2529. The

precursor solution was poured into a mold and cured under UV light. The strain rate (16 mm/s) and interval time (10.8 s) were the same for all tests. Both PVA and GelMa hydrogels achieved milliampere-level output currents; PAAM hydrogel only showed microampere-scale currents. In fact, GelMa and PVA hydrogels do not exhibit high adhesion to ACC; in contrast, PAAM hydrogel, when taken out from the salt solution and wiped, exhibited high adhesion to ACC. The right figure (b) shows the mass change of the ACC electrode before and after contacting the PAAM and PVA hydrogel, respectively. After being pressed against the PAAM hydrogel, the ACC surface is coated with a layer of hydrogel or salt solution, which may affect the surface functionality. The larger output of GelMa hydrogel is probably because it is softer and hence a larger ACC-hydrogel interface area at compressed state than that of the PVA hydrogel. Therefore, the PVA hydrogel was finally selected based on its following merits: (1) PVA hydrogel can be easily obtained with highly consistent mechanical properties through simple freeze-thaw cycles. This allows for better control of variables during the experimental process. (2) by increasing the solid content and the number of freeze-thaw cycles, PVA hydrogel with higher mechanical properties can be obtained. This is beneficial for the demolding process of the hydrogel and helps maintain its resilience, morphology, structure, and stable mechanical properties during subsequent compression-separation testing, enabling it to withstand higher pressures. (3) PVA hydrogel obtained through freeze-thaw cycles can be conveniently assembled, facilitating seamless integration with electrode carbon cloth. (4) the relatively simple and stable chemical structure of PVA is advantageous for analyzing the device performance.

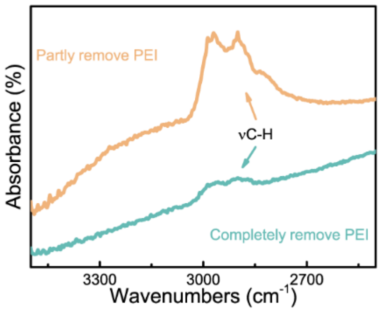

Supplementary Fig. 13 Enlarged FTIR spectrum image, showing that there still exists a small amount of residual PEI on the surface of ACC even after rising for 2 days. As a typical cationic polymer with a strong positive surface charge, PEI is prone to adsorbing negative ions and

repelling positive ions. Due to the abundant negatively charged groups, PEI can be adsorbed on the surface of ACC. In this way, we change the surface charge status from negative to positive, endowing it with the ability to absorb anions.

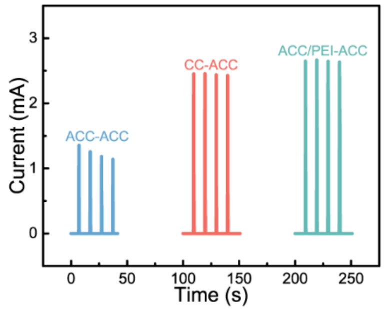

Supplementary Fig. 14 Response currents of three devices with different counter electrodes at ~47 KPa.

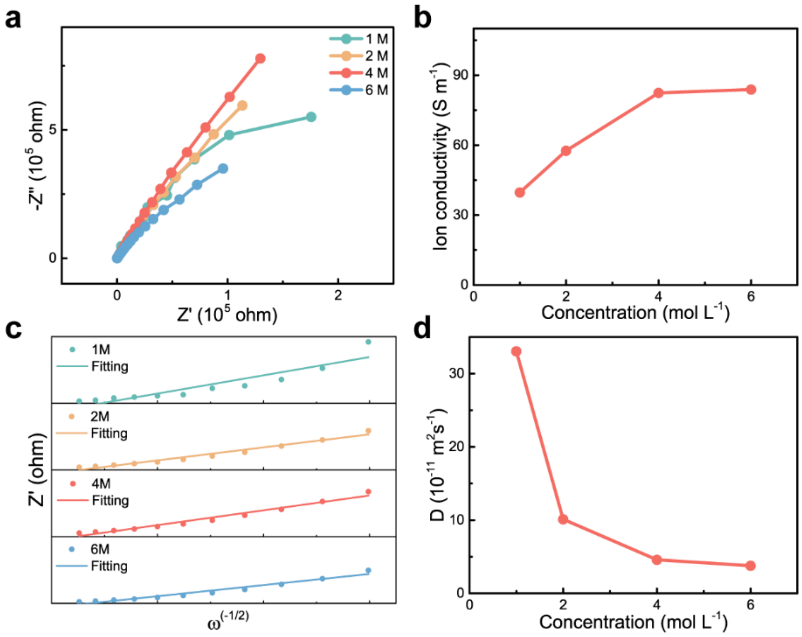

Supplementary Fig. 15 Electrochemical characterization of hydrogel electrolytes. **a,** EIS curves of the PVA hydrogel infused with different concentrations of LiCl. **b,** Ion conductivities of PVA hydrogel infused with different concentrations of LiCl. **c,** Relationship between Z' and $\omega^{-1/2}$. **d,** Diffusion coefficients calculated from the EIS data. The measured ion conductivities and diffusion coefficients align well with reported data[4,5,6,7].

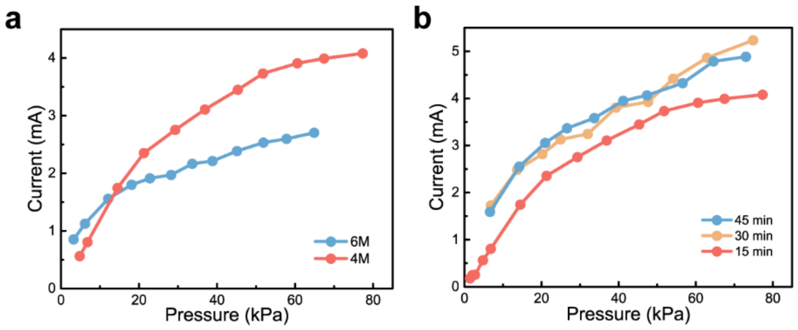

Supplementary Fig. 16 Current outputs from devices with various processing conditions. **a**, Devices with various ionic concentrations. **b**, Devices with various activation time. The device exhibits the highest output performance when the concentration of lithium chloride is 4M. Further increasing the concentration of lithium chloride causes the PVA hydrogel to become sticky, resulting in a decrease in electrical output. Extending the activation time to 30 minutes can enhance the output current. However, during the electrical activation of ACC, the activation current significantly decreases after approximately 17 minutes. Therefore, samples with activation times of 30 minutes and 45 minutes exhibit similar output currents.

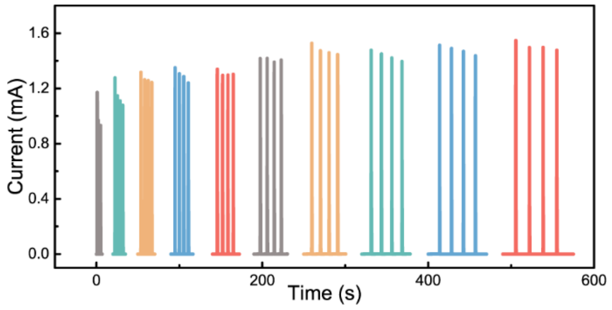

Supplementary Fig. 17 Output current under various recovery times. Longer recovery time (lower frequency) generates more stable and higher currents, which may be attributed to the transient distribution of ions.

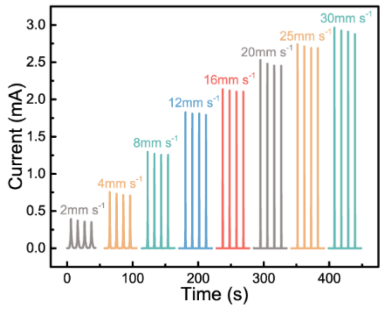

Supplementary Fig. 18 Output current under various strain rates. Higher strain rate results in a

higher peak current. After removing the external pressure, it takes a certain time for ions to return to the initial distribution state. Higher strain rates generate more significant deformation of the hydrogel in a short time, leading to a higher concentration gradient and, thus, higher output current. Meanwhile, higher strain rates shorten the period for ion redistribution, and lead to lower amount of transferred charge per cycle.

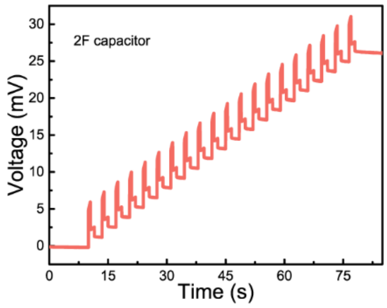

Supplementary Fig. 19 Charging a 2 F capacitor by an electrochemical workstation. In order to simulate the current pulse generated by the device, charging parameters were set as followings: 4 mA for 0.5 s, 1 mA for 1 s, and 0 mA for 2 s. Voltage fluctuation is observed, as mentioned in Fig. 3d, which may be attributed to the influence of the internal resistance of the capacitor.

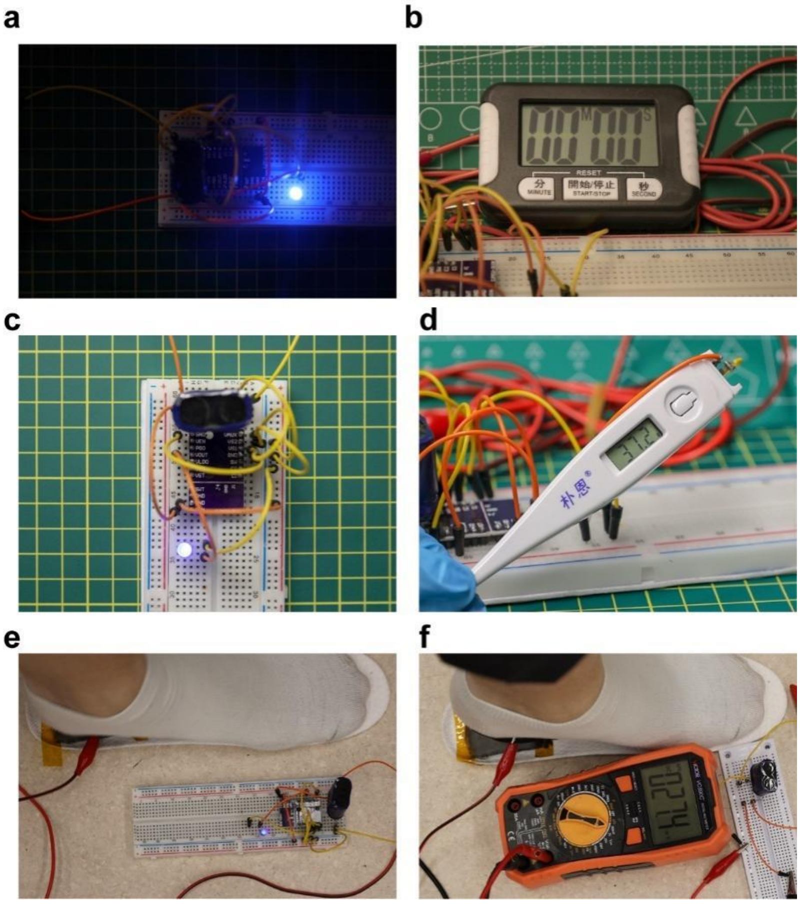

Supplementary Fig. 20 Applications as a power supply to harvest mechanical energy and drive electronics. **a, c**, Powering of a LED. **b**, Powering of a timer. **d**, Powering of a thermometer. **e, f**, Power generation by stepping on the device.

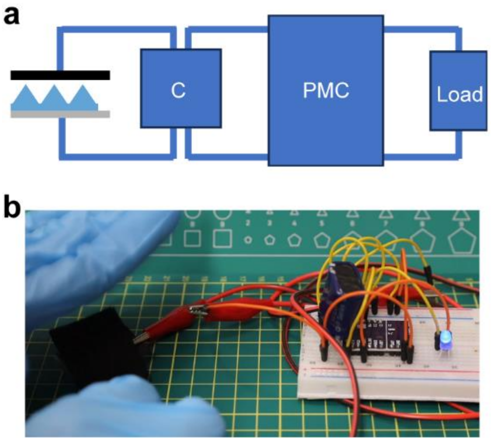

Supplementary Fig. 21 **a,** Equivalent circuit of the power system (C: capacitor; PMC: power management chip, CJMCU 3108) **b,** Photos of the generator connected to the circuit.

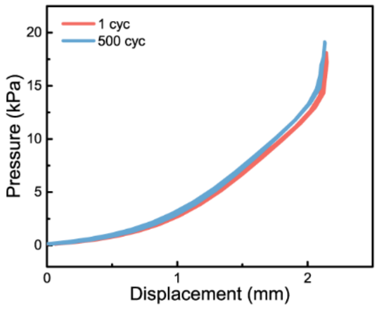

Supplementary Fig. 22 Pressure-distance curve of the 1st and 500th compression-separation cycle. Highly overlapping curves indicate the mechanical stability of the device.

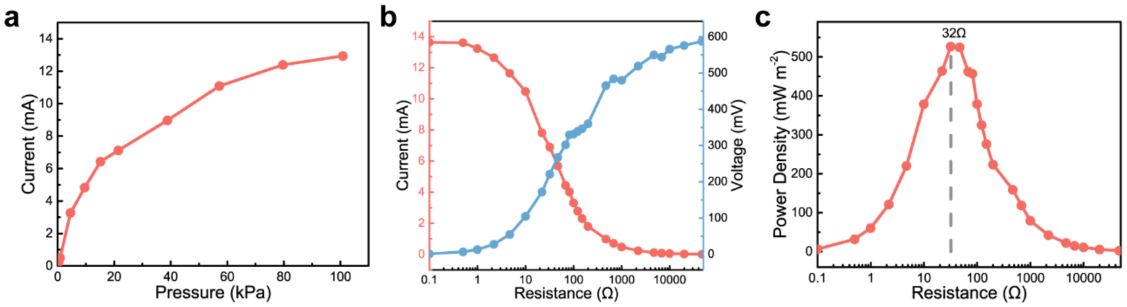

Supplementary Fig. 23 Electrical output performance of the larger-scale device (29 cm^2). **a,** Pressure-response characteristics of the large-scale device. **b,** Current and voltage when loading different resistance. **c,** Power density as a function of external resistance. The change in the area can explain the decrease in internal resistance. Since the thickness and composition of this large-scale device are similar to the previous device, it is reasonable to assume the same resistivity for both. Thus, $R_{large} = R_{small} \times \frac{S_{small}}{S_{large}} = 122 \times \frac{7.29}{29}\ \Omega = 31\ \Omega$, which is close to the measured value of 32 Ω.

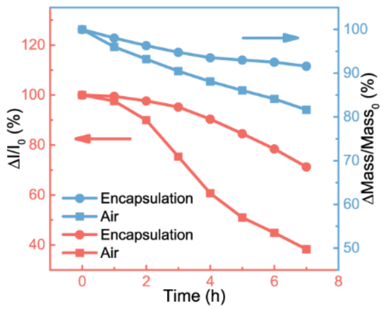

Supplementary Fig. 24 Dehydration of hydrogel energy converters in the atmosphere. One sample was encapsulated with a layer of Parafilm, while the other sample was exposed directly

to the air at a temperature of 27°C and relative humidity of 59%. The mass change is represented by the blue curves, while the red curves depict the output change. It is evident that encapsulation can improve the stability. But future efforts are still required to fully solve this issue.

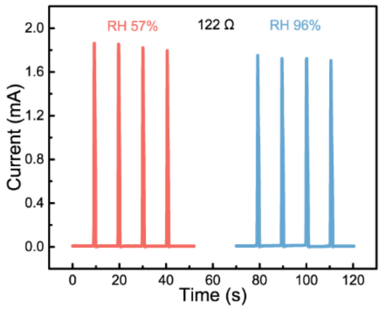

Supplementary Fig. 25 Current signals when loading 122 Ω resistance under low and high humidity, showing that the electrical output is not sensitive to the external humidity.

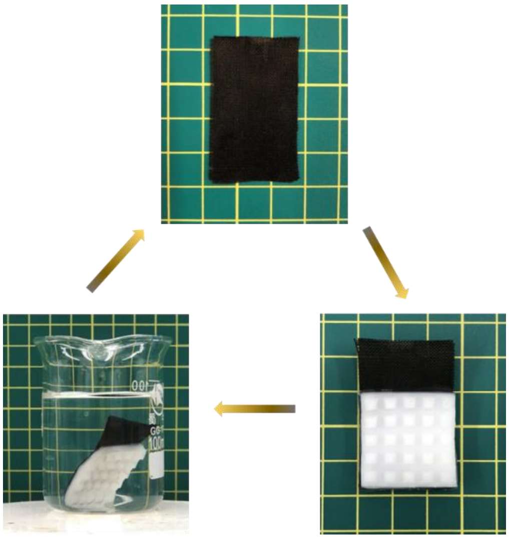

Supplementary Fig. 26 Recycling process of the CC-PVA electrode. Putting the counter electrode into hot water can dissolve the physical-crosslinking PVA hydrogel, recycling the carbon cloth and PVA.

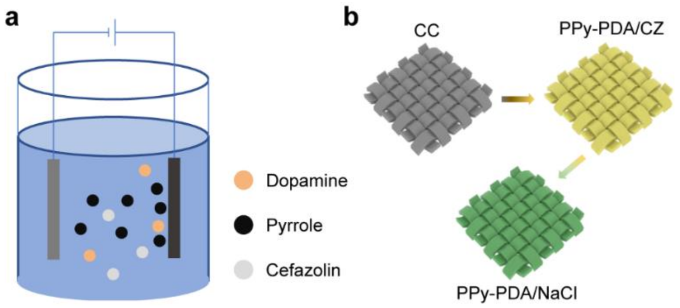

Supplementary Fig. 27 A schematic of drug-loading process with electrodeposition. **a**, Electrochemical deposition. **b**, Deposition sequence for the materials components.

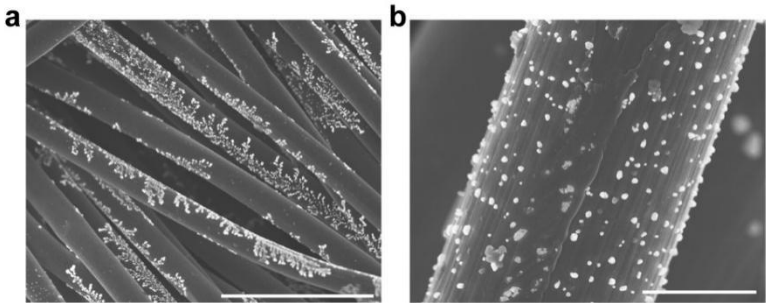

Supplementary Fig. 28 SEM images of CC-CZ. Scale bar: **a,** 50 μm and **b,** 5 μm.

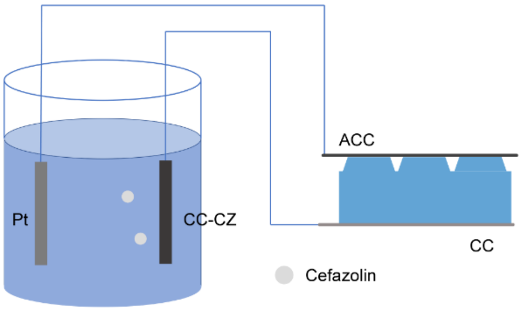

Supplementary Fig. 29 A schematic of the electro-responsive drug release in vitro.

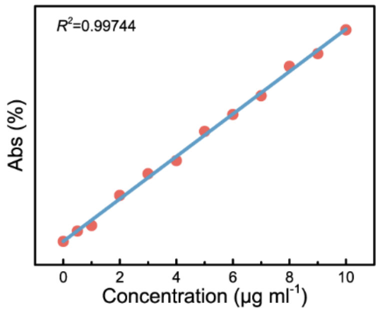

Supplementary Fig. 30 Standard calibration curve of cefazolin sodium.

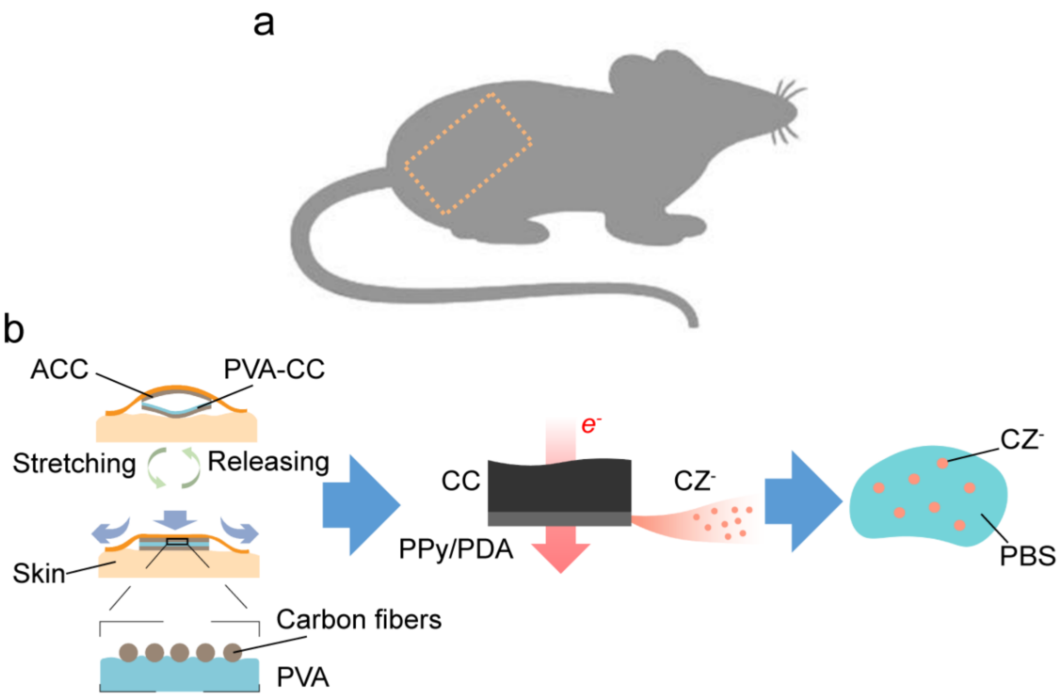

Supplementary Fig. 31 **a,** A schematic showing application of the patch on the dorsal flank side of a mouse. A wound incision was generated under the indicated area. **b,** Schematic image to show the work mechanism of the developed SAP. The soft and biocompatible components allow the device to cover the wound directly. Movements of the mouse, including eating, breathing, etc., stretches the band-aid and causes the band-aid to be flattened, deforming the arched hydrogel, increasing the contact area with the working electrode. Carbon fibers of the carbon cloth induce the uneven deformation of the PVA hydrogel, generating an electric current. The elastic arched PVA hydrogel recovers its shape after stress relaxation, leading to the separation of ACC and PVA.

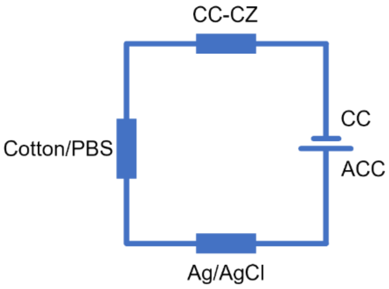

Supplementary Fig. 32 Equivalent circuit of the SAP. The current flows from the ACC

electrode, through the Ag/AgCl circuit, the PBS-containing cotton cloth, and the drug-laden CC-CZ, and back to the CC electrode.

Supplementary Tables

Supplementary Table 1 Comparison with other mechanical-electric energy converters.

Type	Output	Current	Power	Resistance	Loading rate	Form factor	Charging rate	Charge transfer	Ref.
		mA cm^{-2}	W m^{-2}	Ω	Hz; cm s^{-1}		mC s^{-1}	mC m^{-2}	
PENG	AC	0.00064	0.113	6000000	2;-	Soft	0.0294	0.147	8
	AC	0.00071	0.0653	1E+07	1;-	Soft	1.07	3.6	9
	AC	0.00073	0.0605	7E+07	2;-	Soft	0.0396	0.495	10
TENG	AC	-	0.001	4000000	5; 100	Soft	-	-	11
	AC	0.0017	0.328	7000000	1.5; 20	Soft	0.0619	0.0516	12
	AC	0.00179	1.59	1E+07	2;-	Soft	-	-	13
	AC	0.0765	142.5	800000	4;-	Flexible	15.7	3.53	14
	AC	0.0025	10.5	200000	-;-	Flexible	1	0.05	15
SDC-TENG	DC	0.006	0.00015	620	#1; 50	Hard	-	-	16
	DC	0.25	11.85	160000	#2; 50	Hard	96.5	136.3	17
MEG	AC	4.27	20.17	30	20;-	Soft, Weight	3.3	8.25	18
Piezonionic	DC	0.011	0.00085	500	0.1-1;-	Soft	-	800	19
This work	DC	0.55	0.475	122	0.05-1; 0.2-3.3	Soft, Light	159.7	916	

driving method: pressing and sliding. #1: pressure 50 kPa; #2: pressure 30 kPa

Supplementary Table 2 Parameters for the FEA model.

	Mechanical properties	Geometry (mm)
Steel Plate	E=200 GPa, v=0.33	Length=6, width=6, height=0.5
Pyramid Hydrogel	E=100 kPa, v=0.46	Cubic part: Length=6, width=6, height=0.5
		Pyramid part: bottom 4×4, top 0.4×0.4, height 3
Cubic Hydrogel	E=100 kPa, v=0.46	Length=6, width=6, height=3.5

Supplementary Table 3 Parameters used in tests and demonstrations.

		Electrolyte	Electrodes	Recovery time	Strain rate	Pressure
Fig. 1	d, e	4 M LiCl	CC-ACC	10.4 s	16 mm s^{-1}	80 kPa
Fig. 2	a	1 M LiCl	-	10.4 s	16 mm s^{-1}	47 kPa
	b	1 M LiCl	CC-ACC	10.4 s	16 mm s^{-1}	80 kPa
	f	1 M LiCl	-	10.4 s	16 mm s^{-1}	12 kPa
Fig. 3	a	-	CC-ACC	10.4 s	16 mm s^{-1}	-
	b	1 M LiCl	CC-ACC	-	16 mm s^{-1}	17 kPa
	c	1 M LiCl	CC-ACC	10.4 s	-	34 kPa
	d	4 M LiCl	CC-ACC	1.87 s	16 mm s^{-1}	75 kPa
	e	4 M LiCl	CC-ACC	1.28 s	16 mm s^{-1}	5 kPa
Fig. 4	b	4 M LiCl	CC-ACC	-	16 mm s^{-1}	80 kPa
	c	4 M LiCl	CC-ACC	hand	hand	hand

				tapping	tapping	tapping
	d	4 M LiCl	CC-ACC	-	-	-
Default		4 M LiCl	CC-ACC	10.4 s	16 mm s^{-1}	80 kPa

-: variable or not applicable.
Recovery time: Time interval between pressure peaks in two cycles.

Supplementary references

1. Guo, W. et al. Energy Harvesting with Single-Ion-Selective Nanopores: A Concentration-Gradient-Driven Nanofluidic Power Source. *Adv. Funct. Mater.* **20**, 1339-1344 (2010).
2. Jiao, Y., Zheng, Y., Jaroniec, M. & Qiao, S.Z. Origin of the Electrocatalytic Oxygen Reduction Activity of Graphene-Based Catalysts: A Roadmap to Achieve the Best Performance. *J. Am. Chem. Soc.* **136**, 4394-4403 (2014).
3. Finney, A.R., McPherson, I.J., Unwin, P.R. & Salvalaglio, M. Electrochemistry, ion adsorption and dynamics in the double layer: a study of NaCl(aq) on graphite. *Chem. Sci.* **12**, 11166-11180 (2021).
4. Valente AJM, Polishchuk AY, Lobo VMM, Geuskens G. Diffusion coefficients of lithium chloride and potassium chloride in hydrogel membranes derived from acrylamide. *European Polymer Journal* **38**, 13-18 (2002).
5. Yang J*, et al.* Antifreezing Zwitterionic Hydrogel Electrolyte with High Conductivity of 12.6 mS cm^{-1} at −40 °C through Hydrated Lithium Ion Hopping Migration. *Advanced Functional Materials* **31**, 2009438 (2021).
6. Tanaka K, Nomura M. Measurements of tracer diffusion coefficients of lithium ions, chloride ions and water in aqueous lithium chloride solutions. Journal of the Chemical Society, Faraday Transactions 1: Physical Chemistry in Condensed Phases 83, 1779-1782 (1987).
7. Singh MB, Dalvi VH, Gaikar VG. Investigations of clustering of ions and diffusivity in concentrated aqueous solutions of lithium chloride by molecular dynamic simulations. *RSC Advances* **5**, 15328-15337 (2015).
8. Ye, S. et al. High-performance piezoelectric nanogenerator based on microstructured P(VDF-TrFE)/BNNTs composite for energy harvesting and radiation protection in space. *Nano Energy* **60**, 701-714 (2019).
9. Zhao, C., Niu, J., Zhang, Y., Li, C. & Hu, P. Coaxially aligned MWCNTs improve performance of electrospun P(VDF-TrFE)-based fibrous membrane applied in wearable piezoelectric nanogenerator. *Composites Part B* **178**, 107447 (2019).
10. Su, H. et al. Enhanced energy harvesting ability of polydimethylsiloxane-BaTiO3-based flexible piezoelectric nanogenerator for tactile imitation application. *Nano Energy* **83**, 105809 (2021).
11. Jia, L. et al. Electricity Generation and Self-Powered Sensing Enabled by Dynamic Electric Double Layer at Hydrogel–Dielectric Elastomer Interfaces. *ACS Nano* **15**, 19651-19660 (2021).

12. Pu, X. et al. Ultrastretchable, transparent triboelectric nanogenerator as electronic skin for biomechanical energy harvesting and tactile sensing. *Sci. Adv.* **3**, e1700015 (2017).
13. Xu, W. et al. Environmentally Friendly Hydrogel-Based Triboelectric Nanogenerators for Versatile Energy Harvesting and Self-Powered Sensors. *Adv. Energy Mater.* **7**, 1601529 (2017).
14. Wu, H. et al. Achieving Remarkable Charge Density via Self-Polarization of Polar High-k Material in a Charge-Excitation Triboelectric Nanogenerator. *Adv. Mater.* **34**, 2109918 (2022).
15. Wang, H.L., Guo, Z.H., Zhu, G., Pu, X. & Wang, Z.L. Boosting the Power and Lowering the Impedance of Triboelectric Nanogenerators through Manipulating the Permittivity for Wearable Energy Harvesting. *ACS Nano* **15**, 7513-7521 (2021).
16. Zhang, Z. et al. Tribovoltaic Effect on Metal–Semiconductor Interface for Direct-Current Low-Impedance Triboelectric Nanogenerators. *Adv. Energy Mater.* **10**, 1903713 (2020).
17. Zhang, Z. et al. Semiconductor Contact-Electrification-Dominated Tribovoltaic Effect for Ultrahigh Power Generation. *Adv. Mater.* **34**, e2200146 (2022).
18. Zhou, Y. et al. Giant magnetoelastic effect in soft systems for bioelectronics. *Nat. Mater.* **20**, 1670-1676 (2021).
19. Dobashi, Y. et al. Piezoionic mechanoreceptors: Force-induced current generation in hydrogels. *Science* **376**, 502-507 (2022).
